# Supplementary material for: Identification of photoperiod-induced specific miRNAs in the adrenal glands of Sunite sheep (Ovis aries)
Source: Front Vet Sci. 2022 Jul 22;9:888207. doi: 10.3389/fvets.2022.888207 (PMC9354845; doi:10.3389/fvets.2022.888207)
Supplement: Supplementary file 1 [file Data_Sheet_1.ZIP › Supplementary file/miRDeep2 file.zip!/miRDeep2 file/Know_Structure/SP42A1/oar-mir-17.pdf]

miRBase precursor : oar-mir-17
Total read count : 3962
oar-miR-17-5p read count : 3925
remaining reads : 37

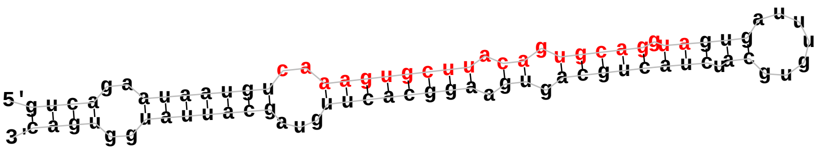

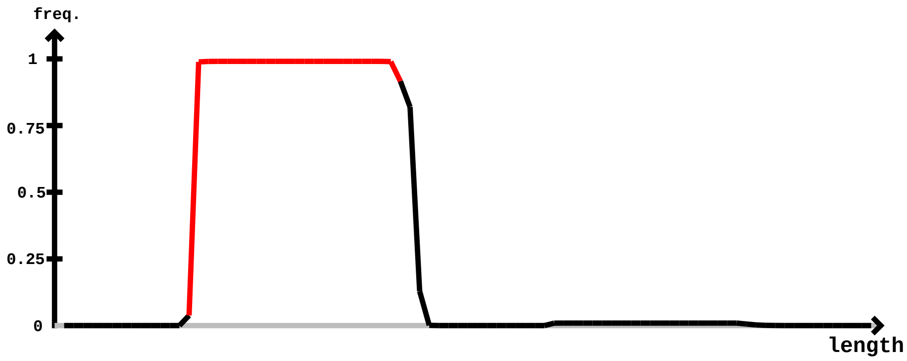


```
                     oar-miR-17-5p
5'- gucagaauaaugucaaagugcuuacagugcagguagugauuugugcaucuacugcagugaaggcacuuguagcauuauggugac   -3'    exp
    ((((..(((((((..((((((((.((.(((((.(((((.......)).)))))))).)).))))))))...)))))))..))))    reads      mm         sample
    ............ucaaagugcuuacagugcagg...................................................    2          0          seq
    ............ucaaagugcuuacagugcaggu..................................................    29         0          seq
    ............ucaaagugcuuacagugcaggua.................................................    25         0          seq
    ............ucaaagugcuuacagugcagguag................................................    89         0          seq
    ............ucaaagugcuuacagugcagguagu...............................................    5          0          seq
    .............caaagugcuuacagugcagg...................................................    2          0          seq
    .............caaagugcuuacagugcaggu..................................................    262        0          seq
    .............caaagugcuCacagugcaggua.................................................    3          1          seq
    .............caaagugcuuacagugcaggua.................................................    353        0          seq
    .............caaagugcuCacagugcagguag................................................    27         1          seq
    .............caaagugcuuacagugcagguag................................................    2617       0          seq
    .............caaagugcuuacagugcagguagu...............................................    495        0          seq
    .............caaagugcuCacagugcagguagu...............................................    5          1          seq
    .............caaagugcuuacagugcagguagug..............................................    3          0          seq
    ..............aaagugcuuacagugcaggu..................................................    1          0          seq
    ..............aaagugcuuacagugcagguag................................................    3          0          seq
    ..............aaagugcuuacagugcagguagu...............................................    2          0          seq
    ..............aaagugcuuacagugcagguagG...............................................    1          1          seq
    ...............aagugcuuaUagugcagguag................................................    1          1          seq
    ..................................................acugcagugaaggcacuugu..............    14         0          seq
    ..................................................acugcagugaaggcacuugua.............    13         0          seq
    ..................................................acugcagugaaggcacuuguag............    7          0          seq
    ..................................................acugcagugaaggcacuuguagc...........    1          0          seq
    ..................................................acugcagugaaggcacuuguagca..........    1          0          seq
    ...................................................cugcagugaaggcacuuguagc...........    1          0          seq
```
